# The Genome of Mycobacterium Africanum West African 2 Reveals a Lineage-Specific Locus and Genome Erosion Common to the *M. tuberculosis* Complex

**DOI:** 10.1371/journal.pntd.0001552

**Published:** 2012-02-28

**Authors:** Stephen D. Bentley, Iñaki Comas, Josephine M. Bryant, Danielle Walker, Noel H. Smith, Simon R. Harris, Scott Thurston, Sebastien Gagneux, Jonathan Wood, Martin Antonio, Michael A. Quail, Florian Gehre, Richard A. Adegbola, Julian Parkhill, Bouke C. de Jong

**Affiliations:** 1 Wellcome Trust Genome Campus, Wellcome Trust Sanger Institute, Hinxton, United Kingdom; 2 Genomics and Health Unit, Centre for Public Health Research, Valencia, Spain; 3 Division of Mycobacterial Research, MRC National Institute for Medical Research, The Ridgeway, Mill Hill, London, United Kingdom; 4 TB Research Group, Veterinary Laboratories Agency (VLA), Weybridge, New Haw, Addlestone, Surrey, United Kingdom and The Centre for the Study of Evolution, University of Sussex, Brighton, United Kingdom; 5 Department of Medical Parasitology and Infection Biology, Swiss Tropical and Public Health Institute, Basel, Switzerland; 6 University of Basel, Basel, Switzerland; 7 Vaccinology Theme, MRC Unit, Banjul, The Gambia; 8 Institute of Tropical Medicine, Antwerp, Belgium; 9 New York University, New York, New York, United States of America; University of Tennessee, United States of America

## Abstract

**Background:**

*M. africanum* West African 2 constitutes an ancient lineage of the *M. tuberculosis* complex that commonly causes human tuberculosis in West Africa and has an attenuated phenotype relative to *M. tuberculosis*.

**Methodology/Principal Findings:**

In search of candidate genes underlying these differences, the genome of *M. africanum* West African 2 was sequenced using classical capillary sequencing techniques. Our findings reveal a unique sequence, RD900, that was independently lost during the evolution of two important lineages within the complex: the “modern” *M. tuberculosis* group and the lineage leading to *M. bovis*. Closely related to *M. bovis* and other animal strains within the *M. tuberculosis* complex, *M. africanum* West African 2 shares an abundance of pseudogenes with *M. bovis* but also with *M. africanum* West African clade 1. Comparison with other strains of the *M. tuberculosis* complex revealed pseudogenes events in all the known lineages pointing toward ongoing genome erosion likely due to increased genetic drift and relaxed selection linked to serial transmission-bottlenecks and an intracellular lifestyle.

**Conclusions/Significance:**

The genomic differences identified between *M. africanum* West African 2 and the other strains of the *Mycobacterium tuberculosis* complex may explain its attenuated phenotype, and pave the way for targeted experiments to elucidate the phenotypic characteristic of *M. africanum*. Moreover, availability of the whole genome data allows for verification of conservation of targets used for the next generation of diagnostics and vaccines, in order to ensure similar efficacy in West Africa.

## Author Summary

*Mycobacterium africanum*, a close relative of *M. tuberculosis*, is studied for the following reasons: *M. africanum* is commonly isolated from West African patients with tuberculosis yet has not spread beyond this region, it is more common in HIV infected patients, and it is less likely to lead to tuberculosis after one is exposed to an infectious case. Understanding this organism's unique biology gets a boost from the decoding of its genome, reported in this issue. For example, genome analysis reveals that *M. africanum* contains a region shared with “ancient” lineages in the *M. tuberculosis* complex and other mycobacterial species, which was lost independently from both *M. tuberculosis* and *M. bovis*. This region encodes a protein involved in transmembrane transport. Furthermore, *M. africanum* has lost genes, including a known virulence gene and genes for vitamin synthesis, in addition to an intact copy of a gene that may increase its susceptibility to antibiotics that are insufficiently active against *M. tuberculosis*. Finally, the genome sequence and analysis reported here will aid in the development of new diagnostics and vaccines against tuberculosis, which need to take into account the differences between *M. africanum* and other species in order to be effective worldwide.

## Introduction

*Mycobacterium africanum* causes up to half of human TB in West Africa and displays differences in patient characteristics and immunoepidemiological features with *M. tuberculosis*, as reviewed earlier in this journal [1]. First described in 1968 in Dakar, Senegal [2], *M. africanum* used to be classified using biochemical methods, until unambiguous classification became possible using molecular methods and two different lineages were identified, *M. africanum* West African type 1, common to Eastern West Africa, and *M. africanum* West African type 2 , common to Western West Africa [3]. Additionally, it became clear that the former “East African *M. africanum*” is genetically part of *M. tuberculosis sensu stricto* [4]. The prevalence of *M. africanum* varies within West Africa, with the highest prevalence of *M. africanum* West African 2 identified in Guinea Bissau (51%, [5]) and the highest prevalence of – West African 1 recorded in Benin (around 28%, [6]). While comparisons between prior prevalence estimates based on biochemical speciation and current estimates based on molecular speciation deserve caution, the prevalence of *M. africanum* appears to be decreasing in Cameroon [7] and Senegal (unpublished results). Comparisons between patients infected with *M. africanum* West African 2 and *M. tuberculosis* suggest that *M. africanum* is somewhat attenuated in its ability to cause disease in immunocompetent hosts [8] and is more common in HIV co-infected patients relative to *M. tuberculosis* in The Gambia [9], yet not in Ghana [10]. Moreover, patients infected with *M. africanum* West African 2, as well as their household contacts, are less likely to mount an IFNg response to ESAT-6 than those infected with *M. tuberculosis* [11]. These two types of *M. africanum*, West African 1 and West African 2, are distinct sub-species within the *M. tuberculosis* complex although it has been suggested that these clades are better described as ecotypes of the *M. tuberculosis* complex rather than sub-species [12]. *M. africanum* West African type 2 is phylogenetically closer to the animal strains like *M. bovis* , with which it shares deletions RD7, 8 and 10 [13], [14], [15], although an animal reservoir for *M. africanum* West African type 2 has not been detected [16]. Subtractive hybridization of *M. africanum* West African type 1 and type 2 from *M. tuberculosis* H37Rv revealed shared and unique genomic differences [3], yet these experiments were not designed to identify regions present in *M. africanum* but absent from *M. tuberculosis*. Here we take advantage of available genomic information for strains of the *Mycobacterium tuberculosis* complex from different sequencing platforms to present and analyze the first complete *M. africanum* West African type 2 genome, that of clinical isolate GM041182 (here designated *M. africanum* GM041182 in the remainder of this manuscript), detailing a novel lineage-defining deletion and an array of species-specific pseudogenes.

## Materials and Methods

### Isolate

*Mycobacterium africanum* GM041182 was isolated in The Gambia in 2004 from a 27 year old HIV uninfected male patient with 3+ smear positive pulmonary tuberculosis. This patient provided written informed consent for participation in the TB Case Contact cohort study, which had been approved by the joint Gambia Government/MRC ethics committee. Moreover, the same ethics committee approved genotyping of bacteria isolated from the patients enrolled in this cohort, and the data were analyzed anonymously. Primary isolation was done in an automated liquid culture system (Bactec MGIT 960, BD) and drug susceptibility testing for first line drugs on solid medium identified no resistance. Genomic DNA was extracted from a single colony sub-culture using the CTAB method [17] and genotyped using spoligotype analysis [18] and PCR for Large Sequence Polymorphism RD702 [3].

### Genome sequencing

The genome of *Mycobacterium africanum* GM041182 was sequenced to approximately 10-fold shotgun coverage, comprising 92612 end sequences, from pOTW12 (with insert sizes 3–4 kb) and pMAQ1Sac_BstXI (with insert sizes of 4–5 kb and 5–6 kb) genomic shotgun libraries using big-dye terminator chemistry on ABI3730 automated sequencers. End sequences from large insert Fosmid libraries in pCC1Fos with an average insert size of 38–42 kb provided scaffold information with approximately 0.2-fold coverage from 2077 end sequences. A 454 FLX sequencing run provided approximately 10-fold single-end shotgun coverage, comprising 224,378 end sequences from 3kb DNA fragments. In addition an Illumina GAII sequencing lane provided approximately 50-fold single-end shotgun sequence, comprising 6083237 end sequences from 200–300 bp fragments and 37 cycles of sequencing. All repeat regions and gaps were bridged by read-pairs or end-sequenced polymerase chain reaction (PCR) products again sequenced with big dye terminator chemistry on ABI3700 capillary sequencers. The sequence was manipulated to the ‘Finished’ standard [19] and is deposited in EMBL/Genbank/DDBJ under accession number FR878060.

### Annotation and genome comparisons

Coding sequences were initially identified by using Glimmer3 [20] and then manually curated using Frameplot [21] and Artemis [22]. All genes were annotated in Artemis using standard criteria [23]. Genome comparisons were visualized in the Artemis comparison tool [24]. Sequence clustering and analysis was performed by using ClustalX 2.0 [25] and MEGA4 [26].

### Genome-based phylogeny of the *M. africanum* GM041182 isolate

To corroborate the phylogenetic position of the GM041182 isolate within the MTBC we took advantage of the availability of Illumina GAIIx runs for different clinical strains representative of the MTBC [27]. We mapped reads for each strain to the genome of GM041182 using MAQ [28] and single nucleotide polymorphisms were called as described in Comas et al. 2010 [27]. A total of 9,699 positions were identified to vary in at least one strain after exclusion of positions with heterozygous calls or deletions (no coverage positions). A phylogeny was inferred using the number of nucleotide differences between strains as the distance measure and Neighbour-joining as the reconstruction method, and 1,000 bootstrap pseudo-replicates were performed to assess the reliability of the clades. Alternative molecular evolution models and phylogenetic methods were not carried out, as a similar set of strains was extensively analyzed before and no difference in topology was observed between the different approaches [27]. All the phylogenetic analyses were carried out using MEGA5 package [29].

### Identification of pseudogenes

A two step process was carried out to identify mutations that either led to the pseudogenization of previously described genes or generated new potential CDS in lineages of the MTBC. Because the genomes of *M. tuberculosis* H37Rv and *M. bovis* AF2122/97 were completed by shotgun sequencing and their annotation manually curated we used them to infer a first list of candidate pseudogenes when compared to *M. africanum* GM041182. CDS were designated as pseudogenes if they contain in the alignment of homologous positions between the three strains either a frameshift or nonsense mutation, were truncated by a deletion event, or interrupted by a large insertion event. As a second step we focus on the microevolution of the MTBC by assessing whether the events leading to truncated or novel CDS were shared among strains of the different lineages of the complex. We took advantage of the availability of draft shotgun sequences of strains belonging to the different lineages (see Supplementary Table S1 for a list of strains and sources). The polymorphisms were corroborated in other strains by blast searches and manual inspection of the alignments. To assign evolutionary directionality to the changes we used as an outgroup the *M. canettii* genome (accession number HE572590).

## Results

### General genome features and phylogenetic position of the GM041182 isolate

General features of the *M. africanum* GM041182 genome are unremarkable relative to other members of the *M. tuberculosis* complex with a typical %G+C content (65.6%) and a genome size (4,389,314 bp) between the usual values for *M. bovis* (4.34–4.37 Mbp) and *M. tuberculosis* (4.40–4.42 Mbp). The *M. africanum* GM041182 genome is also collinear with those of *M. bovis* and *M. tuberculosis* and shares the majority of coding sequences (CDSs). Identification of CDSs present in *M. bovis* and *M. tuberculosis* but absent from strains of *M. africanum* has been presented in several publications to date so will not be further detailed here [3], [30], [31]. However, the availability of the *M. africanum* GM041182 genome sequence has enabled the search for *M. africanum*-specific sequences and the identification of *M. africanum*-specific pseudogenes.

**Figure 1:**
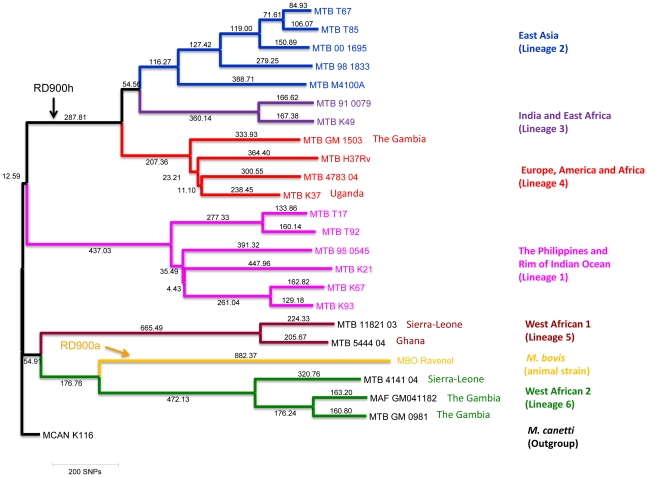
Position of GM041182 in the *Mycobacterium tuberculosis* complex genome phylogeny. The phylogenetic tree is based on the mapping of Illumina data for representative strains of the complex to the newly generated *M. africanum* GM041182. The phylogeny was reconstructed using Neighbor-joining and is based on 9,699 variable positions in at least one strain. One thousand bootstrap pseudo-replicates were used to assess clade reliability. All nodes had more than 90% support. The positions of RD900h and RD900h are indicated. Numbers in branches refers to the corresponding number of SNPs inferred. Lineage names are according Hersbergh 2008 while numbers are according to Comas *et al.* 2010. A review of the nomenclature and comparison with other typing systems can be found in Coscolla and Gagneux 2010 [70] and Comas e*t al.* 2009 [71].

We took advantage of the publicly available Illumina sequencing data for 23 strains representative of the MTBC including the sequences of two lab-adapted strains, *M. tuberculosis* H37Rv and *M. bovis* Ravenel, as well as the sequence of a strain classified as *M. canetti* which we used as an outgroup. We mapped the Illumina short-reads to the newly generated *M. africanum* GM041182 and called for high-confidence polymorphisms. After exclusion of those SNP calls falling in PE/PPE genes and in phage-related regions of the genomes we used an alignment of 9,699 ‘core’ SNP calls (positions in the genome of *M. africanum* GM041182 where at least one strain has a SNP and no strain has a putative deletion or heterozygous call). The resulting phylogeny (Figure 1) placed *M. africanum* GM041182 as part of the *M. africanum* West-African 2 clade (also known as Lineage 6), clustered closely to another strain originally isolated in The Gambia (GM0981). The phylogeny also reflects the great diversity of human *M. tuberculosis* complex strains found in West African countries with circulating strains from at least three different lineages; the two *M. africanum* clades and different sub-lineages belonging to the Euro-American lineage (Lineage 4) which are thought to be recently re-introduced in Africa, as the Euro-American lineage is supposed to have originated in the European region [14].

### Lineage-specific pseudogenes

It has been proposed that due to historical migrations and the low-infectious dose during aerosol transmission of human tuberculosis the effective population size of the bacilli could be reduced. This phenomenon could lead to increased genetic drift, limiting the removal of detrimental mutations through natural selection. Relaxed selection can also act during adaptation to a new niche on those genes for which a selective advantage for maintenance is lost; alternatively, the gene function has become disadvantageous in the new niche. Through base-level inspection of the genome sequences we identified pseudogenes in *M. africanum* GM041182 and verified pseudogene annotation in *M. tuberculosis* H37Rv and *M. bovis* AF2122/97. We identified 120 pseudogenes across the three genomes (*M. africanum* GM041182, *M. tuberculosis* H37Rv, *M. bovis* AF2122/97); 20 were in PE-PGRS/PPE family CDSs and in insertion sequence element transposase genes. Both PE-PGRS/PPE family CDSs and insertion sequence elements are known to be associated with intra-genomic recombination and are susceptible to gene disruption [32], [33], [34].

**Figure 2:**
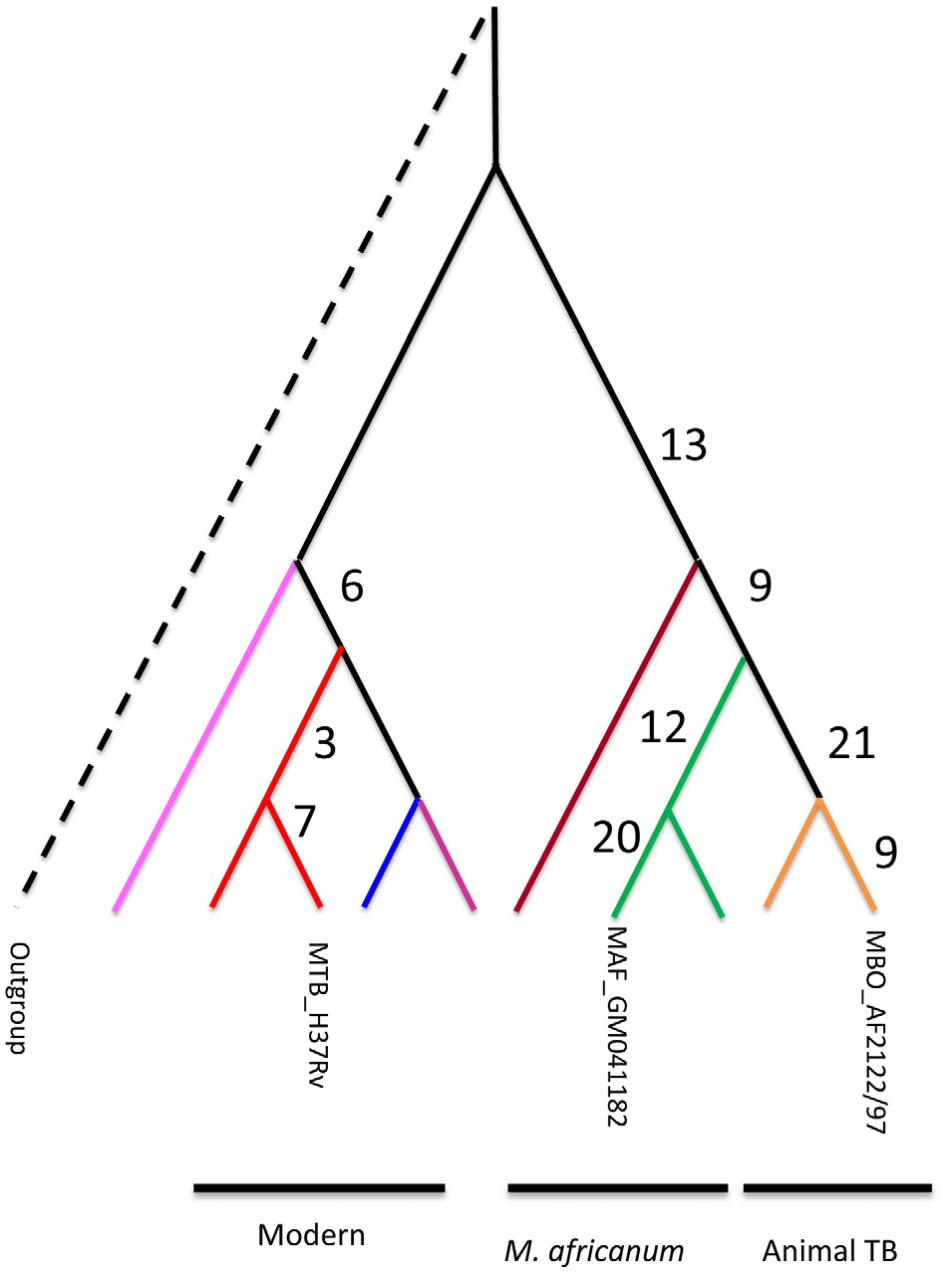
Number of pseudogene events that occurred in the different lineages of the *M. tuberculosis* complex. As the initial number of events was inferred from a three way comparison (see text for details) the number observed is a subset of all possible events that have happened in the evolution of the MTBC. Colours in the figure represent the different lineages of the MTBC as defined in Figure 1. Green: *M. africanum* West African 2 (Lineage 6), Brown: *M. africanum* West African 1 (Lineage 5), Red: *M. tuberculosis* Euro-American (Lineage 4), Purple: *M. tuberculosis* India and East Africa (Lineage 3), Blue: *M. tuberculosis* East Asia (Lineage 2), Pink: *M. tuberculosis* The Philippines and Rim of Indian Ocean (Lineage 1).

We compared the remaining candidate pseudogenes with available draft genomes from different strains belonging to the MTBC lineages and the genome sequence of a *M. canetti* strain as outgroup. By using an outgroup we could determine the genotype of the most likely common ancestor of the MTBC for the different candidate pseudogenes and determine which ones were shared by other strains apart from *M. africanum* GM041182, *M. tuberculosis* H37Rv, *M. bovis* AF2122/97 (Figure 2, Supplementary Table S2). We found that some of the pseudogenes identified were strain-specific occurring only in one of these three strains (20 in GM041182, 7 in H37Rv and 9 in *M. bovis*). More importantly, some of the pseudogene mutations were shared by a large group of strains. For example, 12 were common to the *M. africanum* West-African clade 2 and 13 common to both *M. africanum* clades.

### Gene disruptions target redundant functions

**Figure 3:**
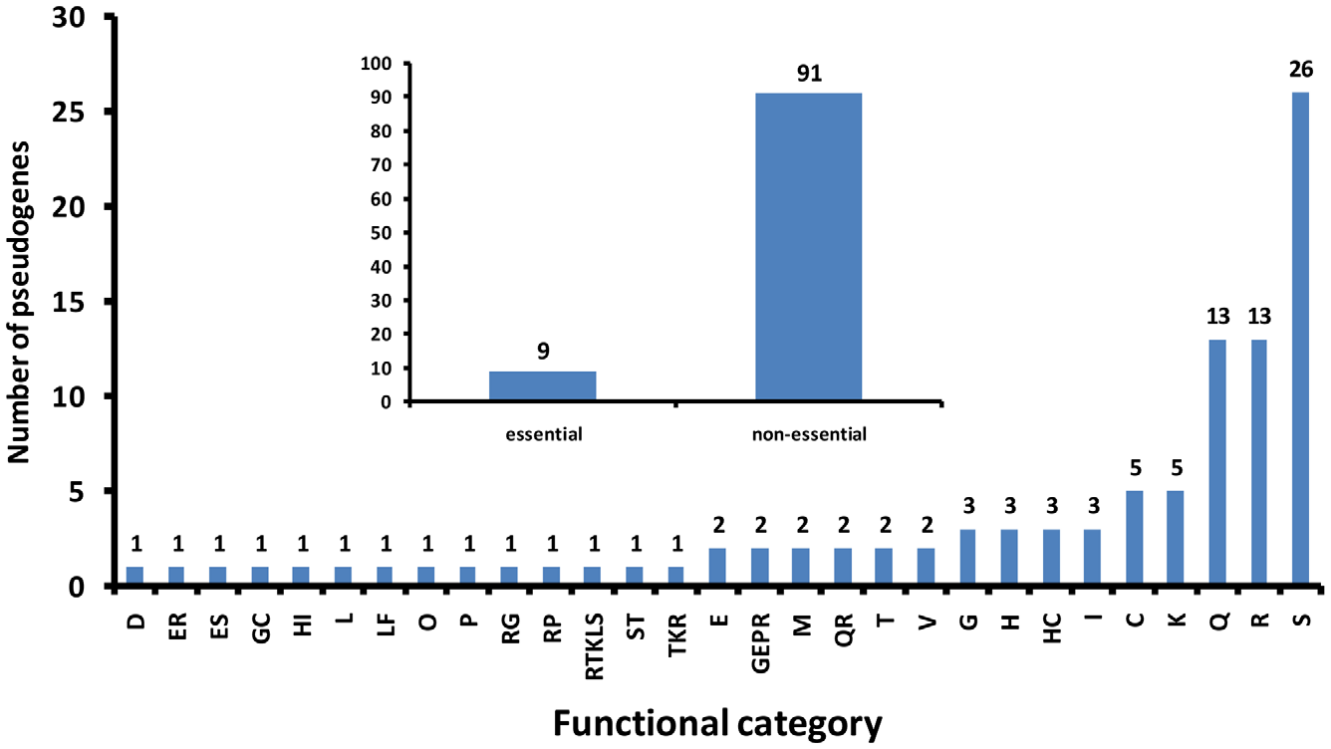
Distribution of the number of pseudogene events by functional category. Clusters of Orthologous Groups (COG) categories were derived from the NCBI *M. tuberculosis* H37Rv annotation while essential/non-essential classification was derived from experiments on transposon mutagenesis [72], [73].

In terms of function, the majority of pseudogenes are hypothetical proteins (N = 39), PE-PGRS family proteins and phage-related (20), metabolic enzymes (13) and transcriptional regulators (5) (Figure 3). Many seem to affect systems which are likely to have functional redundancy due to the presence of paralogous or analogous genes or pathways in the genome. For example, there are three pathways for trehalose biosynthesis in mycobacteria [35]; *M. africanum* and *M. bovis* each have a trehalose biosynthesis pseudogene but are affected in different genes. *M. bovis treY*, encoding maltooligosyltrehalose synthase, has a frameshift due to an internal 806bp deletion while *M. africanum* has a nonsense mutation in a gene (MAF20180) which has been shown to encode a trehalose-phosphate phosphatase. As a component of cell-wall glycolipids, trehalose has been implicated in host tissue damage [35]. Another example is the P450 family of enzymes: there are 21 in the genome of *M. africanum* GM041182, two of them, MAF35300 and MAF31280, are disrupted in all *M. africanum* strains while another (MAF22860), which has been shown to be essential for viability in *M. tuberculosis*, was identified in *M. africanum* GM041182 [36]. Furthermore, of the 10 loci containing at least one polyketide synthase gene, one is disrupted in GM041182, another is disrupted in all West-African 2 clade strains, and another in West-African 2 and animal strains.

The mycobacterial MmpL-family of proteins have a function in lipid transport and have been shown to contribute to *M. tuberculosis* intracellular survival [37]. Both clades of *M. africanum*, as well as animal strains, carry the same nonsense mutation in the 3′ end of the *mmpL12* gene (MAF15490) and in *M. bovis* the *mmpL1* gene (MAF04040) has a central frameshift. These mutations may be predicted to impair lipid transport function, although the presence of 12 *mmpL* paralogues per genome implies some degree of redundancy. Another redundant system affected by mutation in *M. bovis* is the so-called mammalian cell entry (*mce*) operons. *M. bovis* has two adjacent pseudogenes (*mce2D* and *mce2E*) in one of the four *mce* (mammalian cell entry) operons. In *M. tuberculosis*, deletion of the *mce2* operon attenuates the ability to infect mice [38], and deletion of more than one *mce* operon has a cumulative effect indicating non-redundant roles during infection [39].

The ability to metabolize nitrate to nitrite is thought to be important for *M. tuberculosis* to persist under anaerobic conditions during dormancy and also appears to have functional redundancy [40]. *M. africanum* GM041182 has a pseudogene relevant to nitrate metabolism (*narX*) and all *M. africanum* and animal strains harbor a *narU* pseudogene. Although the majority of nitrate reductase activity *in vitro* is due to *narGHJI* [41], *narX*, which encodes a fusion protein equivalent to parts of *NarG*, *NarJ* and *NarI*, has also been shown to have a role in dormancy [40]. NarU is thought to be involved in transport of nitrate into and nitrite out of the bacterial cell though again its function is thought to be secondary to that of the more active *narK2* which coincidentally is adjacent to *narX*.

*M. africanum* West-African clade 2 strains have frameshift mutations in one of the 17 adenylate cyclase genes in the genome (MAF03880). In *M. tuberculosis* the MAF03880 orthologue (*Rv0386*) was recently found to produce a cyclic AMP burst within macrophages that influences cell signaling. Loss of *Rv0386* resulted in lower TNF-a induction, decreased immunopathology in animal tissues, and diminished bacterial survival [42].

Three genes with a role in drug efflux have been disrupted; one in *M. africanum* GM041182 strain (MAF03440), one in *M. bovis* (orthologue of MAF18990) and one in all the so-called ‘modern’ MTBC strains (MAF23460). The isoniazid inducible gene, *iniA* (MAF03440), thought to be involved in an efflux pump for two of the 1^st^ line TB drugs, isoniazid and ethambutol [43] has a 5′ nonsense mutation in *M. africanum* GM041182. In *M. tuberculosis* an *iniA* deletion mutant showed increased susceptibility to isoniazid [43], suggesting that *M. africanum* may be more susceptible to isoniazid than *M. tuberculosis*. This mutation is however not present in other *M. africanum* strains of which the genome is available, nor in clinical isolates of the same lineage originating from Burkina Faso and Cote d'Ivoire (data not shown), suggesting that this polymorphism is unique to strain GM041182 . Deletion of the *M. smegmatis* orthologue of MAF18990 has been shown to result in reduced resistance to ethidium bromide, acriflavine and erythromycin [44]. More interesting is the evolution of the MAF23460 gene. Its homologue in H37Rv is Rv2333c. By inspecting the alignment of both genes a single base pair deletion in the H37Rv leads to a longer product than that observed in *M. africanum* GM041182 strain (524 residues in *M. africanum* GM041182 versus 538 residues in *M. tuberculosis* H37Rv). By comparing with the rest of strains of the complex it becomes clear that the single base deletion occurred in the common ancestor of ‘modern’ lineages representing in this case a possible gain of function rather than a pseudogenetization per se of the ancestral genes. Rv2333c has been shown to be involved in export of spectinomycin and tetracycline and thus contributes to the intrinsic resistance of *M. tuberculosis* to these antibiotics [45], which may thus be more effective against *M. africanum*.

Further pseudogenes affect non-redundant systems such as biosynthesis of vitamins B12 (cobalamin) and B6; three genes in a cobalamin biosynthesis operon (MAF20880, MAF20850 and MAF20870) have the same pseudogene allele in both *M. africanum* and *M. bovis* while the *pdxH* (MAF26250) vitamin B6 biosynthesis gene has a central frameshift mutation in *M. africanum* GM041822 strain. Supplementation of these vitamins may support growth of *M. africanum* and reduce the growth delay of *M. africanum* relative to *M. tuberculosis*.

A notable non-redundant pseudogene is the previously identified orthologue of *Rv3879c* (MAF38940), part of the RD1 region [46], that was found to be essential for ESAT-6 secretion, but not CFP-10, in *M. marinum* but not in *M. tuberculosis*. In a recent study using immunoblots for ESAT-6 and control antigens, we found ESAT-6 secretion to be similar between *M. africanum* GM041182, *M. africanum* GM041182 complemented with *Rv3879c*, and *M. tuberculosis* H37Rv [47], which does not corroborate the attenuated ESAT-6 response in *M. africanum* infected people [11]. Given the ESX-1 homology throughout the MTBC it is currently not clear how equal amounts of secreted ESAT-6 between *M. africanum* GM041182 and *M. tuberculosis* H37Rv can correlate with the attenuated ESAT-6 response observed in *M. africanum* infected people. Ongoing immunoepidemiological analyses however suggest that the attenuated ESAT-6 phenotype may cluster with sub-lineages within the *M. africanum* West African 2 lineage. In addition, we identified a deletion in the upstream regulatory region of *Rv3616c* whose expression is related with ESAT-6 secretion [48]. This polymorphism in GM041182 is shared with animal strains, in which it is responsible for decreased expression of *Rv3616c* (Roger Buxton, personal communication). Interestingly, ESAT-6 is highly immunogenic in *M. bovis* infected cows [49], suggesting that the genetic basis for the attenuated ESAT-6 response observed in *M. africanum* infected persons is specific to *M. africanum*.

Finally, the *M. tuberculosis* orthologue of MAF29630 (Rv2958c) encodes a glycosyl transferase which has been shown, in a co-infection assay, to confer increased resistance to killing by human macrophages [50]. Both *M. africanum* clades and *M. bovis* have a single base pair insertion that shortens the gene product (367 residues in *M. africanum* GM041182 versus 429 residues in *M. tuberculosis* H37Rv).

### Identification of a lineage-specific sequence - RD900

On comparative genomics of *M. bovis* and *M. tuberculosis* H37Rv, a region unique to *M. bovis* was designated TB deleted 1 (TbD1) [51]. Subsequent work identified the TbD1 deletion to be shared by “modern” *M. tuberculosis* lineages, with an intact TbD1 region in other animal strains, *M. africanum*, and “ancient” *M. tuberculosis* [15]. Proteins from the TbD1 region were however not immunogenic in an ELISPOT assay that aimed to identify lineage specific immune responses [52].

Comparison of the *M. africanum* GM041182 genome with reference shotgun sequences for *M. bovis* (strains BCG Pasteur 1173P2, BCG Tokyo 172, AF2122/97) and *M. tuberculosis* (strains H37Rv, H37Ra, CDC1551, F11) genomes revealed a single region present in *M. africanum* GM041182 but deleted in *M. bovis* and “modern” *M. tuberculosis* strains. This *M. africanum* specific locus was designated RD900. The locus is 3,141 bp long and contains a single complete gene (designated *maf1* (MAF12860)) and the 3′ end of another (MAF12870); *maf1* encodes a putative ATP-binding cassette (ABC) transport protein that has a central ATP-binding domain and six possible membrane-spanning domains in the C-terminal portion. In addition the N-terminal region contains a putative Forkhead associated (FHA) domain that may confer the ability to bind DNA and thereby potentially act as a transcriptional regulator. The LpqY-SugA-SugB-SugC ABC transporter (Rv1235-Rv1238), one of four ABC transporters in *M. tuberculosis*, has recently been characterized as a recycling system mediating the retrograde transport of the sugar trehalose produced and released by the bacterium [53]. Other ABC transport proteins may mediate efflux of drugs and other compounds (Rv1218c, [54]), with implications for immune responses (Rv1280c-Rv1283c, [55]).

Of the 51 proteins in the Pfam database with the same domain architecture as RD900, only twelve are from outside the order Actinomycetales (seven from Cyanobacteria and five from Chloroflexi); none have been experimentally characterised.

**Figure 4:**
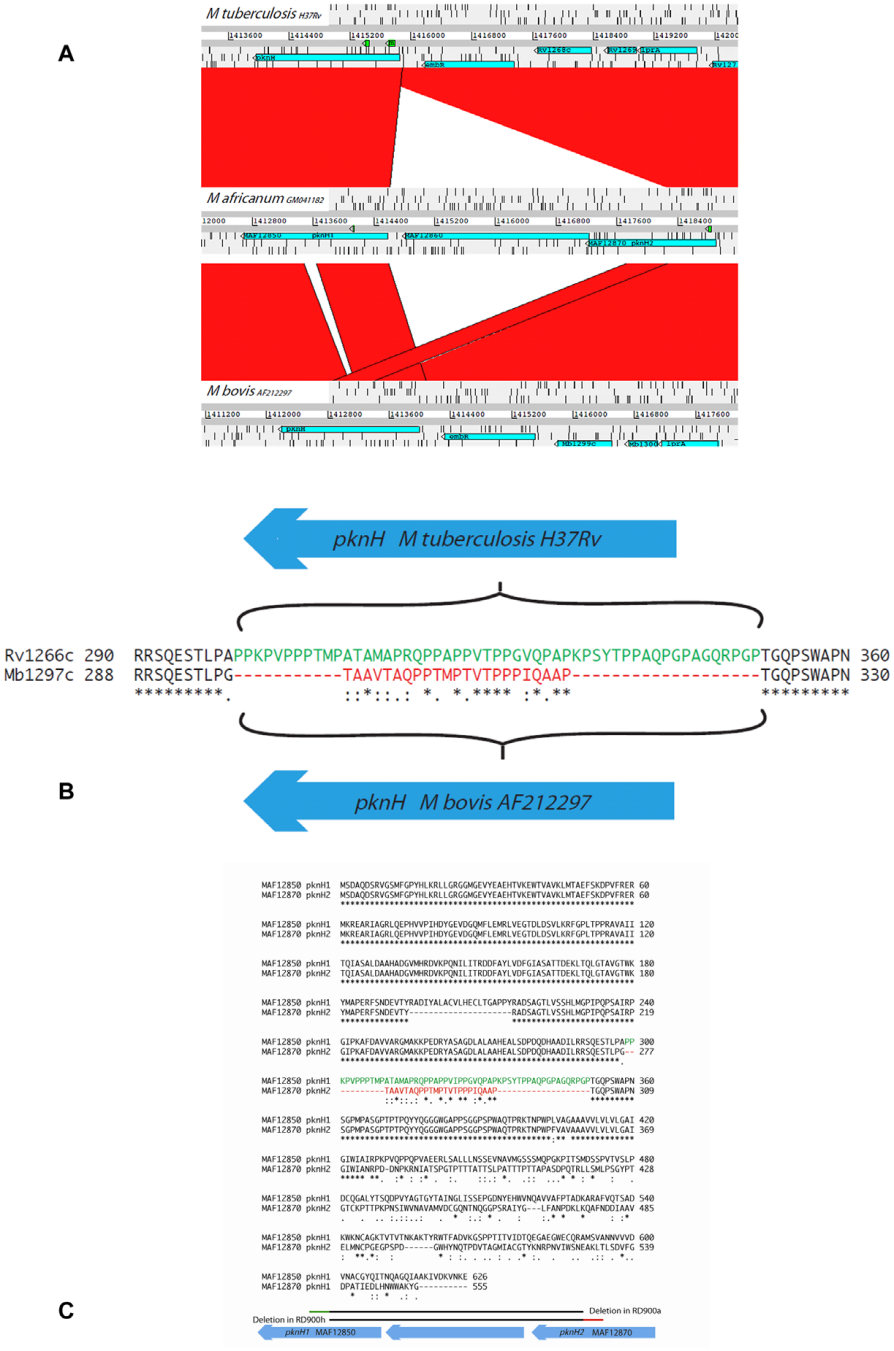
A. Comparison of the RD900 locus in *M. bovis*, *M. africanum* and *M. tuberculosis*. The RD900 deletion is present in *M. bovis* (AF212297)and *M tuberculosis* (H37Rv) identified through a genome comparison with *M. africanum* (GM041182). Figure adapted from use of Artemis Comparison Tool. B. Alignment of PknH1 and PknH2 in *M. africanum* (GM041182). The first two thirds of PknH1 and PknH2 have a high level of sequence identity except for two distinct regions. The first region is an INDEL region from codons 194–214 in PknH1. The second is a substitution region where there are 53 amino acids in PknH1 (green) instead of a region of 23 amino acids in PknH2 (red). The substitution region allows us to identify two different RD900 deletions; RD900h in *M. tuberculosis*, and RD900a in *M. bovis*. C. Alignment of the substitution region of the PknH gene of *M. tuberculosis* and *M. bovis*. A different composite PknH gene has resulted from two different RD900 deletions described in B.

Assuming complete absence of recombination, comparison of the RD900 region of *M. africanum* GM041182 with all available genomes from the *Mycobacterium tuberculosis* complex suggests that intact RD900 (*M. africanum* GM041182) represents the ancestral state of this region and the RD900 region was independently deleted in two lineages: the “modern” *M. tuberculosis* lineage and a sub-branch of the animal associated lineage leading to *M. bovis* (Figure 1). We checked this in two ways. First, we aligned complete genomes available for the MTBC with *M. africanum* GM041182. Secondly, we did local BLAST searches of the region including the two flanking genes (from MAF12850 to MAF12880) (see Figure 4). The very unusual occurrence of independent deletions generating the same functional gene, *pknH*, is explainable when the flanking regions are considered.

In *M. africanum* GM041182, *maf1* is flanked by similar, co-directional genes, each encoding a protein kinase. The RD900 deletion appears to have been generated by recombination between these flanking genes to form *pknH*, a protein kinase-encoding gene found in *M. bovis* and *M. tuberculosis* (Figure 2a), thus *pknH* is apparently a composite gene made up by intra-genomic recombination between two homologous, physically close, genes. Accordingly we have designated the flanking genes in *M. africanum* GM041182 as *pknH1* (downstream) and *pknH2* (upstream). These flanking CDSs in *M. africanum* GM041182 have a high level of amino acid identity for the first two-thirds of their length (Figure 2b) followed by divergent sequences from codon 424 (PknH1) and 373 (PknH2), onwards. However, the homologous regions of PknH1 and PknH2 in *M. africanum* GM041182 have two significant differences. The first is a 21 amino acid sequence insertion/deletion region, present in PknH1 from codons 194 to 214 but absent from PknH2. The second is a substitution region of 53 amino acids in PknH1 (298–350) with low identity to a region of 23 amino acids in PknH2 (277–299). These two differences account for the 50 codon difference in the region of high identity between PknH1 and PknH2. More importantly, the substitution region can be used to demonstrate that the deletion of the *pknH* gene in *M. tuberculosis* (called RD900h, Figure 1) was independent of the RD900 deletion found in *M. bovis* (RD900a, Figure 1). Deletion RD900h generates a composite *pknH* gene in *M. tuberculosis* identical to *M. africanum pknH1* in the substitution region while the RD900a deletion found in *M. bovis* generates a composite *pknH* gene with a substitution region identical to that of *M. africanum pknH2*. This implies that the RD900h and RD900a deletions had end points before and after the substitution region, respectively. The remaining 3′ portion of *pknH* for *M. tuberculosis* and *M. bovis* has a high degree of similarity to *M. africanum pknH1*, consistent with the architecture of the region in *M. africanum*.

The formation of an intact protein kinase in *M. tuberculosis* and *M. bovis*, where deletion could easily have resulted in two non-functional gene fragments, could be used to suggest a selective advantage for the reduction in the number of protein kinases caused by the RD900 deletion. However, this conclusion must be tempered by the ease with which this deletion can be generated. We assume that at least one functional *pknH* gene is required by strains of the *M. tuberculosis* complex so it is not unexpected that extant strains appear to have a functional gene at this locus.

**Figure 5:**
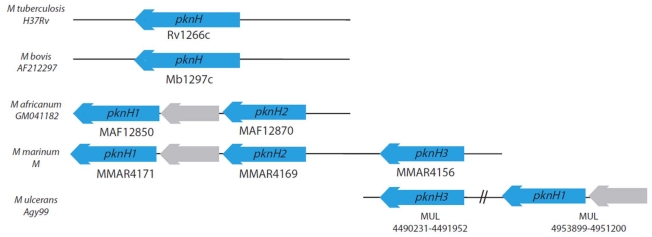
The RD900 locus in other mycobacteria. RD900 region is deleted in both *M. tuberculosis* (H37Rv) and *M. bovis* (AF212297) but not *M. africanum* (GM041182) *M. canetti* (CIPT140010059), *M. marinum* (M) or *M. ulcerans* (Agy99). In *M. marinum* an additional PknH gene is present (designated as PknH3). PknH3 is also present in *M. ulcerans*, where the PknH1 locus and a 5′ CDS (grey) have been duplicated/translocated to somewhere else in the genome (position 4951200–4955915). The entire “ancestral” PknH locus is not present. PknH genes were identified through comparative genome comparison of available shotgun sequences.

Broadening the phylogenetic scope of the analysis to include complete genomes from other members of the genus *Mycobacterium* reveals further patterns of mycobacterial genome evolution associated with RD900. It seems plausible that the ancestral chromosome arrangement for the clade including the *M. tuberculosis* and *M. avium* complexes was similar to that seen in *M. marinum* where an extra 13,772 bp flanks the *pknH2* side of RD900 (Figure 5). This flanking region ends with another protein kinase gene which we designate here as *pknH3* and the *M. tuberculosis* complex ancestral genome may have undergone a deletion due to recombination between *pknH2* and *pknH3*.

The *M. ulcerans* Agy99 genome appears to have undergone the deletion of MURD111 (which removes pknH2) and a long-range rearrangement separating *pknH1* and *maf1* on the left flank from the right hand flank which actually carries *pknH3*.

The genomes of *M. avium* and *M. avium subspecies paratuberculosis* have simple RD900 deletions akin to those seen in the modern *M. tuberculosis* and *M. bovis* lineages; *M. leprae* TN genome has a similar deletion pattern but with extra DNA loss equivalent to the region from 1412285 to 1423476 in *M. africanum* GM041182, which results in the loss of nine genes including *pknH1*, *maf1* and *pknH2*. Curiously, *M. smegmatis* has a similar deletion pattern to *M. leprae* suggesting a convergent event in this distant “rapid growing” relative. Also interesting is the high degree of synteny between *M. africanum* GM041182 and *M. kansasii* ATCC 12478 with equivalent RD900 arrangement and colinearity extending for at least 6 kilobases on both flanks.

## Discussion

The *M. africanum* GM041182 genome is, as expected, highly homologous to those of other members of the *M. tuberculosis* complex, yet contains a unique sequence, RD900, that was independently lost during the evolution of two important lineages within the complex; the “modern” *M. tuberculosis* group and the lineage leading to *M. bovis*. In addition, RD900 is variably present in atypical mycobacteria, with evidence for repeated independent deletion events. We can expect to learn more about the phylogenetic position of this deletion as more mycobacterial genomes are sequenced, with this complete *M. africanum* West African 2 sequence serving as an alternative reference for the mapping of further *M. africanum* genomes generated using Next Generation Sequencing techniques. Determining the function of the deleted gene, *maf1*, and the phenotypic consequences of its deletion will require further study but nevertheless this occurrence may provide valuable insight into the evolution of the complex.

The similarity in pseudogene repertoire suggests that *M. africanum* has a similar evolutionary history to *M. bovis* and it is tempting to speculate that this may have involved adaptation to a non-human animal host, though it must be noted that for both lineages nearly half of the pseudogenes are unique, so subsequent adaptations may have occurred since their divergence reflecting contemporary niche differences. Thus far, no candidate animal reservoir has been detected for *M. africanum*. Extensive searches among cattle, sheep, pigs, and goats in the Gambia and neighbouring countries have not identified mycobacterial infection nor disease [16], [56], [57]. Phylogenetically, the Dassie bacillus [58] and the recently identified *Mycobacterium mungi* [59], are the closest relatives of *M. africanum* within the *M. tuberculosis* complex. The Dassie bacillus has been isolated from Dassies, or Rock Hyrax, in South Africa [60], and *M. mungi* causes disease in troops of banded mongoose in northern Botswana [59]. However, an extensive search for mycobacteria in terrestrial small mammals in Benin, West Africa, did not identify any members of the *M. tuberculosis* complex [61].

The lack of spread of *M. africanum* from West Africa to the Americas at the time of the slave trade remains enigmatic. Today, *M. africanum* is rarely isolated outside of West Africa, typically in first degree immigrants [62]. In a study in Ghana, host polymorphisms were identified with differential protection against *M. tuberculosis* versus *M. africanum* in both directions [63], [64], although the degree of selective advantage conferred by these polymorphisms is unclear.

The majority of the pseudogenes detected are only disrupted by a single base mutation, either by an insertion/deletion leading to a frameshift or by substitution leading to a nonsense mutation. As expected for a recently evolved pathogen no further disrupting mutations have been identified in the pseudogenes. Similarly, in a comparison of several MTBC genomes that included GM041182, no mutations were identified in the promoter region of the pseudogenes, supporting the notion that “pseudogenization” in the MTBC is recent [65]. A formal statistical testing of the rate of acquisition of pseudogenes cannot be carried out because of the bias in the discovery of the pseudogenes described to those observed in the comparison of *M. africanum* GM041182 with the two other strains leading to a phenomenon of pseudogene discovery bias.

However, the high number of pseudogenes in *M. africanum* and other strains of the *M. tuberculosis* complex (MTBC) suggest that genome erosion is ongoing. Most likely this reflects several different phenomena that have lead to the downsizing of the MTBC genomes as compared to other free-living or opportunistic Mycobacteria [66], [67]. This could be partly due to its recent evolution as an intracellular pathogen, making some functions that served a free-living lifestyle redundant to the MTBC, which was therefore prone to lose the function due to relaxed selection. At the same time natural selection can act to favour the loss of some genes. These “anti-virulence genes” can be lost because they can be detrimental for the pathogenic lifestyle as has been described for other species [68] and suggested for some known deletion events in the MTBC [69]. Finally, the increased genetic drift imposed by transmission bottlenecks and changes in population size of its host, lead to a weakened effect of natural selection and increased accumulation of functional mutations, many of them detrimental [14]. Further studies, such as complementing the virulence gene Rv0386 in *M. africanum* and assessing the effect in the appropriate animal model, can assess to which extent its lower progression to disease is explained by these pseudogenes. Moreover, the presence in *M. africanum* GM041182 of the original version of MAF23460 (Rv2333c) without gain of function suggests that *M. africanum* (and other ancestral *M. tuberculosis* complex lineages) lack this functional efflux pump and may be more susceptible to antibiotics, possibly including spectinomycin and tetracycline.

Differentiating these processes by comparative genomics within and outside the complex could provide clues about how the tight relationships between MTBC species and their respective hosts arose in the first place, and how the ongoing erosion described here generates different genetic backgrounds within the complex than can explain some of the differences associated with diversity in disease outcome [70].

## Supporting Information

Table S1 List of genome data sources
(XLSX)

Table S2 Pseudogenes
(XLSX)
